# Sustained improvement of elastosis perforans serpiginosa after isotretinoin therapy

**DOI:** 10.1016/j.jdcr.2024.02.006

**Published:** 2024-02-19

**Authors:** Sasan D. Noveir, Jessica Wu, Kathy Kim Langevin

**Affiliations:** a David Geffen School of Medicine at University of California Los Angeles, Los Angeles, California; b Division of Dermatology, Department of Medicine, David Geffen School of Medicine at University of California Los Angeles, Los Angeles, California; c Wieder Dermatology and Laser Center, Los Angeles, California

**Keywords:** case report, elastosis perforans serpiginosa, isotretinoin, perforating dermatosis, retinoid

## Introduction

Elastosis perforans serpiginosa (EPS) is a rare skin disease characterized clinically by keratotic papules arranged in a serpiginous or annular pattern.^1^ This disorder frequently manifests in young males, predominantly impacting the extremities, face, and neck.^2^ The defining feature is the presence of abnormal elastic fibers protruding through the papillary layer and epidermis.^3^ The etiology of EPS remains unclear, and its incidence is not well-established. EPS can be further classified into idiopathic cases, drug-induced reactions, or those associated with genetic diseases. Managing this condition poses challenges, as there is no universally accepted standard treatment, and many patients fail topical and systemic therapies.^3^ This report describes a case of a young woman who exhibited sustained improvement in her EPS 3 months following completion of isotretinoin therapy for her acne.

## Case report

**Fig 1:**
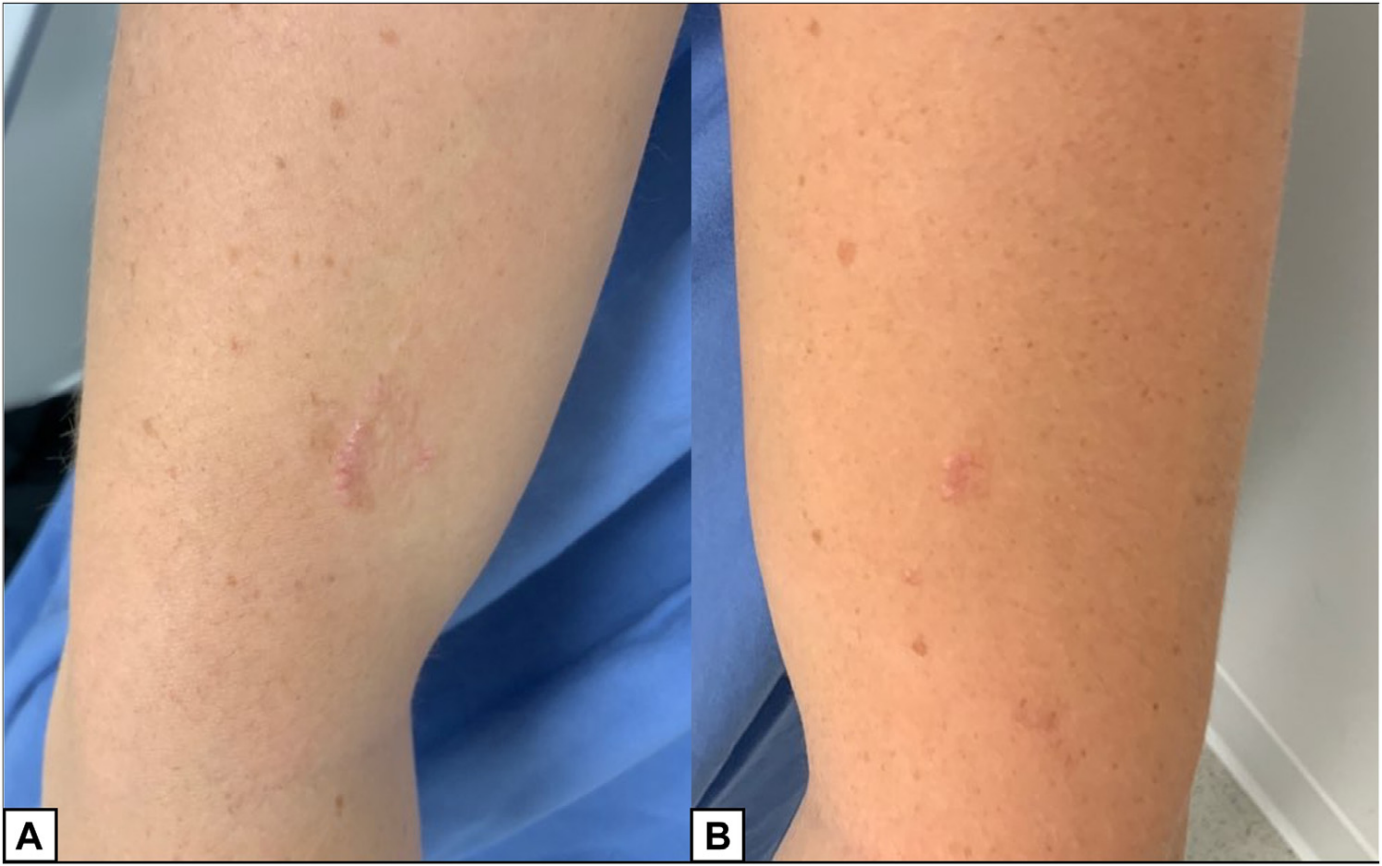
Right upper arm (**A**) and left upper arm (**B**) with EPS on initial presentation. *EPS*, Elastosis perforans serpiginosa.

**Fig 2:**
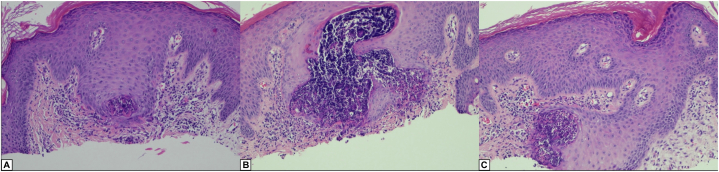
Histopathologic examination at magnification 10× (**A**-**C**) of a representative biopsy of the right upper arm.

A healthy 15-year-old female presented for evaluation of a progressing eruption on her upper arms. The lesion initially appeared on her right upper arm but had since enlarged and extended to her left upper arm over the past 6 months. There was no associated pruritus, but the lesions were occasionally tender. Her medical history was notable for a nevus sebaceous, which was removed at an outside clinic 4 years prior to presenting with EPS. Clinical exam demonstrated clusters of erythematous, keratotic papules coalescing into an arcuate distribution on the upper arms (Fig 1). A biopsy of the right arm lesion demonstrated short eosinophilic elastic fibers penetrating the epidermis and the follicular infundibulae, consistent with EPS (Fig 2). The patient underwent a 2-month trial of triamcinolone 0.1% cream and a 4- month trial of tretinoin 0.05% cream, but both treatments failed to improve the lesions.

**Fig 3:**
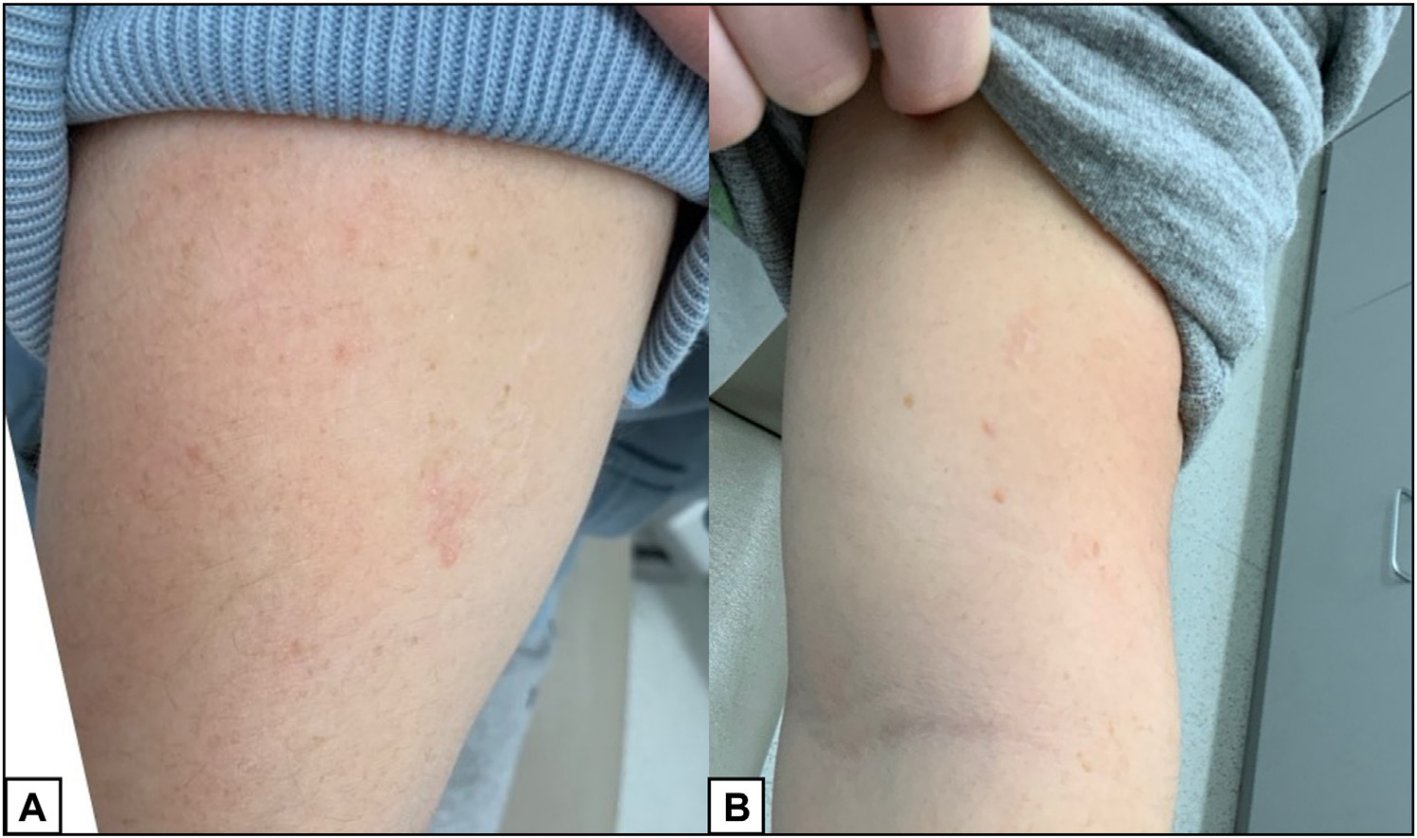
Improvement of EPS on right upper arm (**A**) and left upper arm (**B**) 1 month after initiating isotretinoin. *EPS*, Elastosis perforans serpiginosa.

**Fig 4:**
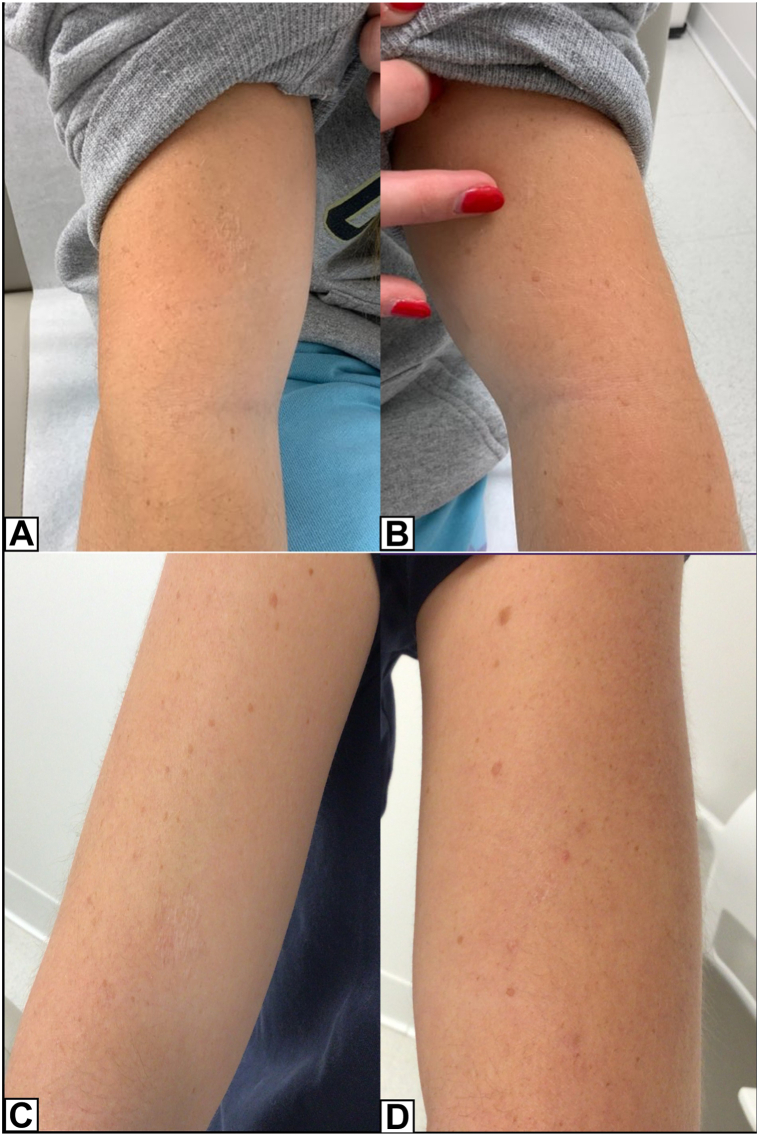
Examination of the right upper arm (**A**) and left upper arm (**B**) after 3 months following isotretinoin course completion. Sustained improvement seen on right upper arm (**C**) and left upper arm (**D**) 6 months posttreatment.

Two years following her initial presentation with EPS, she was started on isotretinoin for acne treatment. The patient was also started on combined ethinyl estradiol and norgestimate pills for contraceptive management while on isotretinoin. After 1 month of isotretinoin 40 mg/day (0.75 mg/kg/day), the patient noticed significant improvement in her EPS (Fig 3). After 6 months, the patient discontinued her oral contraception for nonmedical reasons. Isotretinoin was eventually increased to 50 mg/day (1 mg/kg/day) for a total course of 185 mg/kg completed over 8 months. At her 6-month follow up appointment, she showed sustained improvement in her EPS (Fig 4).

## Discussion

EPS is a rare condition classified as a primary perforating dermatosis. These disorders are characterized by the presence of dermal connective tissue pushing through the epidermis, leading to the formation of papules or nodules.^3^ Approximately 75% of EPS cases occur in males, with the condition typically manifesting in early childhood or young adulthood.^2^ While the majority of cases manifest sporadically in otherwise healthy individuals, as demonstrated in this report, a subset is linked to various genetic disorders and medications. Diseases associated with EPS include Ehlers-Danlos syndrome, Marfan’s syndrome, Down syndrome, and osteogenesis imperfecta.^1^ Drug-induced EPS commonly arises with D-penicillamine use in patients undergoing treatment for Wilson's disease.^4^

EPS typically manifests as keratotic, small papules arranged in an annular or serpiginous pattern, with lesions that are asymptomatic or pruritic. Differential diagnosis may include granuloma annulare, sarcoidosis, skin calcinosis, and tinea corporis. The diagnosis is confirmed by biopsy, which demonstrates elastic fibers arranged in a corkscrew pattern penetrating the epidermis.^3^

Various treatments have been reported for managing EPS with differing degrees of success. However, there is currently no established gold standard therapy for the condition and clinical management relies solely on case reports.

This case reports the second instance of effectively treating EPS with isotretinoin. For a subset of time during isotretinoin therapy, the patient was also prescribed combined ethinyl estradiol and norgestimate pills. However, it is unlikely this contributed to the resolution of her EPS, as there was additional improvement during isotretinoin therapy after she discontinued the contraceptive pills. The first case in the literature documented the management of penicillamine-induced EPS using isotretinoin at a daily dosage of 0.5 mg/kg/day while continuing penicillamine therapy.^5^ Similar to our case, notable improvement was observed after 6 weeks. Subsequently, the patient was transitioned off penicillamine for reasons unrelated to EPS and discontinued isotretinoin. Other cases have reported improvement with 0.1% tazarotene gel; however, the condition flared upon discontinuing the treatment.^6^ Other cases have documented treatment failure with various topical retinoids and oral isotretinoin at dosages ranging from 40 to 60 mg/day for 15 weeks.^6^ This underscores the challenge in treating EPS, as effective medications in one case may not necessarily work for the next patient. Additionally, destructive methods such as cryosurgery, cellophane tape stripping, curettage, and electrocautery, have demonstrated limited success.^2^^,^^6^, ^7^, ^8^ These modalities are also associated with scarring, keloid formation, hyperpigmentation, and atrophy.

Managing EPS poses a challenge, with numerous cases illustrating the ineffectiveness of mechanical destruction, as well as topical and systemic therapies. Despite some reported cases of successful therapeutic interventions, recurrences can still occur spontaneously. Furthermore, reports of spontaneous resolution add a layer of complexity to accurately interpreting therapeutic efficacy.^2^^,^^9^

## Conflicts of interest

None disclosed.
